# Primeira Intervenção Coronária Percutânea de Alto Risco do Brasil com Suporte Hemodinâmico de Dispositivo Pulsátil Percutâneo: Um Relato de Caso

**DOI:** 10.36660/abc.202250236

**Published:** 2025-11-27

**Authors:** André M. Nicolau, Pedro H. M. C. de Melo, Lucas Aguiar Alencar de Oliveira, Rafael Bergo, Thiago A. Kleinsorge, Roger Renault Godinho, Fábio Sândoli de Brito, Alexandre A. Abizaid, Carlos de Magalhães Campos

**Affiliations:** 1 Hospital das Clínicas da Faculdade de Medicina da Universidade de São Paulo Instituto do Coração São Paulo SP Brasil Instituto do Coração do Hospital das Clínicas da Faculdade de Medicina da Universidade de São Paulo, São Paulo, SP – Brasil; 2 Hospital Sírio-Libanês São Paulo SP Brasil Hospital Sírio-Libanês, São Paulo, SP – Brasil

**Keywords:** Intervenção Coronária Percutânea, Coração Auxiliar, Doença da Artéria Coronariana

## Abstract

A intervenção coronária percutânea (ICP) é o método de revascularização coronariana mais utilizada no mundo, mesmo em casos de alta complexidade anatômica. No entanto, a crescente complexidade elevou os riscos associados ao procedimento. Dispositivos de assistência circulatória mecânica (DACMs), como o iVAC 2L, têm sido empregados para suporte hemodinâmico em ICPs de alto risco.

Descrevemos o primeiro caso no Brasil de ICP realizada com suporte do iVAC 2L, um DACM pulsátil. O paciente, um homem de 81 anos, com angina classe funcional III e lesões graves em tronco de coronária esquerda (TCE), descendente anterior (DA) e circunflexa (CX), associada a calcificação extensa. O procedimento foi realizado com suporte do iVAC 2L e realizada ICP com implante de stent em TCE, DA e CX. Todo o procedimento ocorreu sem necessidade de drogas vasoativas e o dispositivo foi removido sem complicações vasculares.

A ICP de alto risco tem se tornado cada vez mais comum, muitas vezes exigindo suporte mecânico ventricular. O iVAC 2L apresenta vantagens como fluxo pulsátil e facilidade de implante, sendo uma alternativa ao balão intra-aórtico e ao Impella. O relato destaca a possibilidade do uso do iVAC 2L no cenário nacional, ampliando as opções terapêuticas para pacientes de alto risco.

## Introdução

A intervenção coronária percutânea (ICP) é o método de revascularização mais realizado, mesmo em casos de alta complexidade anatômica, particularmente em pacientes de alto risco cirúrgico^1,2^ Entretanto, com o aumento da complexidade anatômica, associado ao envelhecimento populacional e aumento da incidência de comorbidades, observa-se um aumento do perfil de risco das ICP.^3–5^

Neste contexto, os dispositivos de assistência circulatória mecânica (DACM) reconhecidamente promovem melhora dos parâmetros hemodinâmicos^6,7^ e são recomendados para suporte circulatório mecânico temporário no contexto de ICP de alto risco.^8–11^

**Figura 1:**
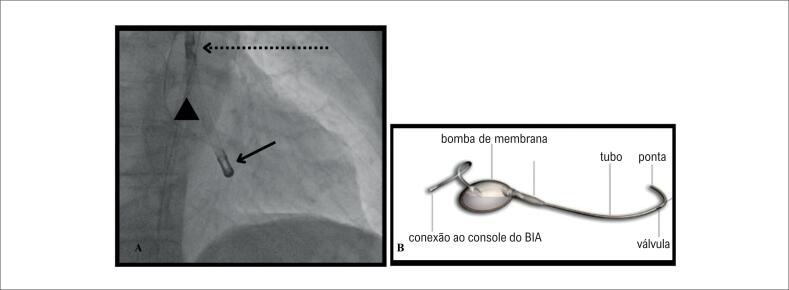
1A) Posicionamento do dispositivo iVAC 2.0 com ponta do ventrículo esquerdo (seta cheia) e válvula direcional na aorta ascendente (seta pontilhada). Fio guia 0.035'auxilia reconhecimento da posição do seio aórtico (marcada com triângulo). 1B) Dispositivo iVAC 2.0.

O dispositivo iVAC 2L (*Pulsecath BV, Amsterdam, The Netherlands)* é um DACM de inserção retrógrada pela artéria femoral. O dispositivo consiste em uma válvula bidirecional conectada a uma membrana extracorpórea por uma cânula de 17Fr. O sangue é aspirado do ventrículo pela ponta do cateter durante a sístole até a membrana e, durante a diástole, a membrana ejeta o sangue na aorta ascendente pela válvula bidirecional (Figura 1) A movimentação da membrana ocorre às custas da mobilização de gás hélio pelo console de balão intra-aórtico (Vídeo 1), que comanda o funcionamento do dispositivo. A ponta do cateter é posicionada retrogradamente abaixo da valva aórtica e dentro do ventrículo esquerdo (Figura 1). Sob condições ideais (frequência cardíaca 70 - 80 bpm) o débito cardíaco adicional é de até 2.0L/min, com fluxo pulsátil. A segurança e o funcionamento do iVAC 2L foram reportadas previamente.^12,13^

**Vídeo 1:**
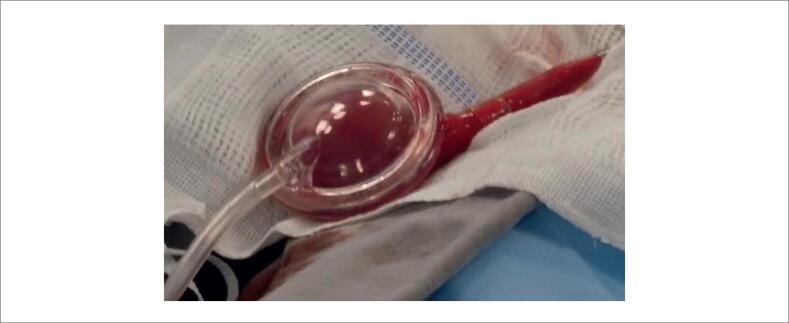
Disponível em: http://abccardiol.org/supplementary-material/2025/12210/2025-0236_RC_Video_1.mp4

Apresentaremos o primeiro caso realizado no Brasil de ICP complexa de alto risco realizada com suporte do dispositivo iVAC 2L.

## Caso Clínico

**Figura 2:**
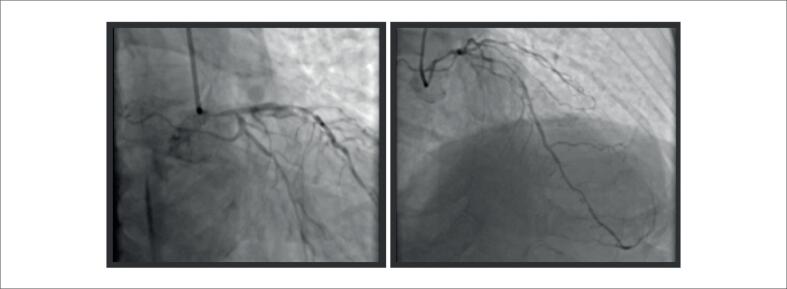
Cateterismo cardíaco demonstrando lesões de 80% em tronco de coronária esquerda (TCE), 80% em DA e 70% em Cx.

Reportamos o caso de um homem de 81 anos, hipertenso e diabético, encaminhado ao nosso serviço por dispneia e angina classe III. Foi realizada avaliação funcional com cintilografia que demonstrou isquemia na parede anterior e anterosseptal, além de queda da pressão arterial no pico do esforço e fração de ejeção do ventrículo esquerdo de 34%. Indicou-se então cateterismo cardíaco, que revelou lesão em tronco de coronária esquerda (TCE) (80%), descendente anterior (DA) (lesões de 70 e 80% em terços proximal e médio) e artéria circunflexa (CX) (70% em terço proximal), associada a extensa calcificação coronária (Figura 2) e Syntax Score 32. O caso foi discutido em "Heart Team" e foi optado por tratamento percutâneo após ponderação de alto risco cirúrgico por Euroscore II 7.74%, STS 5.82% e fragilidade clínica avaliada por equipe multidisciplinar.

**Figura 3:**
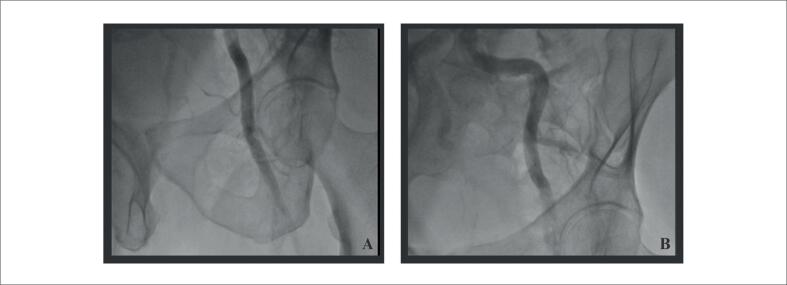
Arteriografia femoral e inguinal esquerdas. Figura 3A: arteriografia femoral esquerda demonstrando bom calibre da via. Figura 3B: arteriografia ilíaca esquerda demonstrando bom calibre da via e importante tortuosidade em ilíaca comum.

O procedimento foi iniciado com punção femoral seguida de arteriografia bilateral (Figura 3) para decisão da melhor estratégia. Ambas as artérias femorais comuns apresentavam calibres adequados para acomodar o introdutor do dispositivo iVAC 2L. Observada lesão aterosclerótica importante no terço distal da femoral comum direita, sendo optado por realizar o posicionamento do dispositivo iVAC 2L pela artéria femoral comum esquerda.

Realizou-se pré-fechamento da artéria femoral esquerda com dois dispositivos *Perclose* proglide e inserção de introdutor 18F hidrofílico, por fio-guia 0,035″ de alto suporte ("*Amplatz Super Stiff")*. Pela artéria femoral direita, utilizou-se um fio-guia 0,035″ posicionado no seio não coronário para marcar o nível da valva aórtica. Na sequência, avançou-se o dispositivo iVAC 2L posicionando sua ponta no ventrículo esquerdo, 2 cm abaixo da valva aórtica, mantendo a válvula bidirecional na aorta ascendente (Figura 1). O dispositivo foi conectado ao console do BIA (balão intra-aórtico), configurado para disparo por ECG, com insuflação máxima e disparo 1:1, fornecendo um débito adicional estimado em torno de 2.0L, visto frequência cardíaca do paciente em torno de 70-80bpm (Vídeo 2).

**Vídeo 2:**
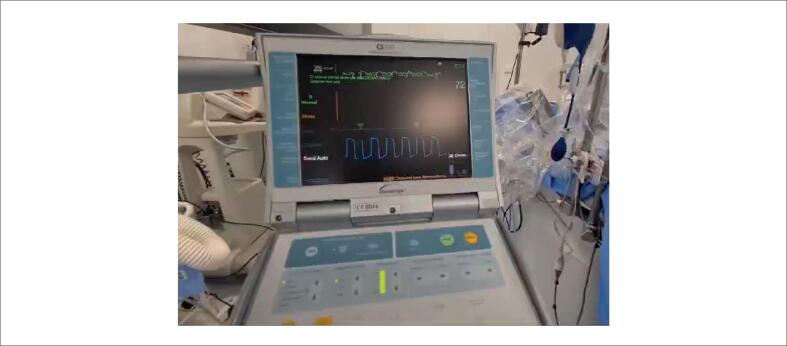
Disponível em: http://abccardiol.org/supplementary-material/2025/12210/2025-0236_RC_Video_2.mp4

**Figura 4:**
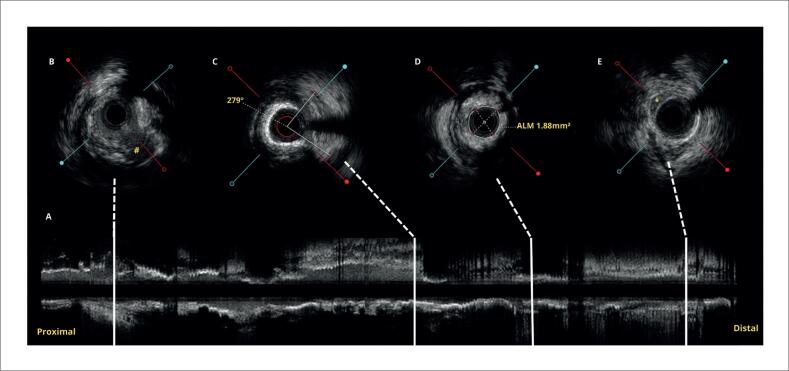
Avaliação com ultrassom intracoronário (USIC) pré implante de *Stent* com fio-guia posicionado em segmento distal da artéria descendente anterior. 4A) corte longititudinal (*Long view*) da coronária descendente anterior até o tronco da coronária esquerda. 4B) presença de úlcera em TCE, marcada com "#", demonstrando comunicação da lesão com o lúmen. 4C) Calcificação extensa em descendente anterior, com ângulo de calcificação >270° reconhecido pela sobra acústica posterior ausente apenas entre 1 e 4 horas. 4D) Área luminal mínima em descendente anterior com 1.88mm^2^. 4E) Presença de ponta miocárdica em porção distal do segmento avaliado (marcado com "*"), servindo como referência distal para posicionamento de Stent. Sinal da "meia-lua" ou *Half-moon sign*.

**Figura 5:**
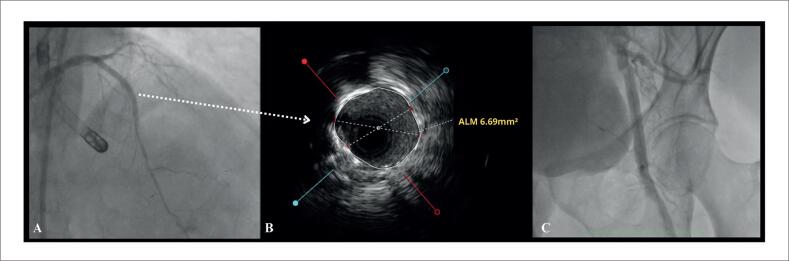
Resultado final angiográfico e ultrassonográfico. 5A) Resultado final com técnica de Mini-Crush TCE-DA e Cx. 5B) Avaliação final de USIC com área luminal mínima intra-stent 6.69mm^2^. Figura 5C: Arteriografia femoral esquerda final sem complicações.

Utilizamos cateter EBU 3.5 7Fr para cateterização da coronária esquerda. Pela presença de lesão gravemente estenótica, realizada pré-dilatação para permitir avaliação com ultrassom intracoronário (USIC), que demonstrou área luminal mínima de 1,88mm^2^, placa fibrolipídica e calcificação importante, além de ulceração em TCE e ponte miocárdica no segmento distal da artéria (Figura 4). Foi realizado, preparo da lesão com balões dedicados (*Cutting Balloon* e balão não complacente*)*, com resultado adequado comprovado por USIC. A seguir, implantamos Stents farmacológicos em DA média, CX ostial com mínima protrusão para o tronco, e DA proximal com extensão para o tronco, conforme técnica de "*Mini-Crush"*. Realizada pós-dilatação dos stents, e ajuste de carina com técnica de "*Kissing-balloon".* Realizado USIC de controle com adequada expansão e aposição das hastes, sem complicações (Figura 5). Todo o procedimento foi realizado sob sedação consciente, sem uso de droga vasoativa. Após o procedimento, o dispositivo foi retirado e realizada hemostasia com perclose, conforme mencionado. A arteriografia final não demonstrou complicações vasculares (Figura 5).^8^

## Discussão

ICPs de alto risco vem se tornando cada vez mais frequentes e com melhores resultados.^3–5^ Neste contexto, diversos DACM foram desenvolvidos.^14^ Historicamente, o BIA tem sido utilizado na prática clínica para suporte mecânico em ICPs de alto risco. A evidência proveniente de ensaio clínico randomizado neste contexto falhou em demonstrar benefício em redução de eventos cardiovasculares maiores intra-hospitalares, apesar de análises de longo prazo apontarem para possível, embora especulativo, benefício de longo prazo.^6,15^ Assim, embora o BIA ofereça suporte mecânico e melhora da perfusão coronária e cerebral, o modesto ganho de débito cardíaco e a falta de evidência de benefícios cardiovasculares levou a sua não recomendação pelas diretrizes e consensos.^2,8^

O funcionamento e segurança do dispositivo iVAC 2L foram demonstrados em estudos prévios,^12,13,16^ fornecendo um débito cardíaco de até 2.0L/min, cerca de 3-4x o débito fornecido pelo BIA (Tabela 1). Quando comparado aos dispositivos de fluxo contínuo como *Impella* e ECMO, apresenta como diferenciais o fluxo pulsátil possivelmente mais fisiológico, a facilidade no implante e o uso de console de balão já disponível em diversos centros, dispensando um console dedicado. Se comparado a ECMO V-A, o iVAC apresenta como vantagem, assim como o Impella e o BIA, a descompressão do ventrículo esquerdo e diminuição da resistência vascular sistêmica. Como desvantagens, destaca-se o perfil de 18F (frente os 14F do Impella CP) e o menor suporte (até 2L frente aos 4.3L do Impella ou até 10L/min da ECMO V-A) (Tabela 1).

**Tabela 1:** Comparação entre os dispositivos iVAC 2L, BIA, Impella e ECMO V-A

	iVAC 2L	BIA	Impella	ECMO V-A
Débito adicional	2,0L/min	0,3-0,5L/min	2,5-5,5L/min	Até 7L/min
Acesso	Femoral 18Fr	Femoral 7-8Fr	13-14Fr	14-19Fr Arterial 17-21Fr ECMO
Mecanismo	Membrana extracorpórea	Contrapulsação	Bomba microaxial	Bomba centrífuga
Sincronia com ritmo cardíaco	Sim	Sim	Não	Não
Pós-carga	Diminui	Diminui	Diminui	Aumenta

## Disponibilidade de Dados

Os conteúdos subjacentes ao texto da pesquisa estão contidos no manuscrito.
