# Supplementary material for: Primeira Intervenção Coronária Percutânea de Alto Risco do Brasil com Suporte Hemodinâmico de Dispositivo Pulsátil Percutâneo: Um Relato de Caso
Source: Arq Bras Cardiol. 2025 Nov 27;122(10):e20250236. [Article in Portuguese] doi: 10.36660/abc.202250236 (PMC12674855; doi:10.36660/abc.202250236)
Supplement: Translation

# Translation: Brazil's First High-Risk Percutaneous Coronary Intervention with Hemodynamic Support from a Percutaneous Pulsatile Device: A Case Report

**DOI:** 10.36660/abc.202250236i

**Authors:** André M. Nicolau, Pedro H. M. C. de Melo, Lucas Aguiar Alencar de Oliveira, Rafael Bergo, Thiago A. Kleinsorge, Roger Renault Godinho, Fábio Sândoli de Brito, Alexandre A. Abizaid, Carlos de Magalhães Campos

**Affiliations:** 1 São Paulo SP Brazil Instituto do Coração do Hospital das Clínicas da Faculdade de Medicina da Universidade de São Paulo, São Paulo, SP – Brazil; 2 São Paulo SP Brazil Hospital Sírio-Libanês, São Paulo, SP – Brazil

**Keywords:** Percutaneous Coronary Intervention, Heart-Assist Devices, Coronary Artery Disease

## Abstract

Percutaneous coronary intervention (PCI) is the most widely used coronary revascularization method worldwide, even in cases of high anatomical complexity. However, this increasing complexity has increased the risks associated with the procedure. Mechanical circulatory assist devices (MCADs), such as the iVAC 2L, have been used for hemodynamic support in high-risk PCIs.

We describe the first case in Brazil of PCI performed with the support of the iVAC 2L, a pulsatile MCAD. The patient, an 81-year-old man, had functional class III angina and severe lesions in the left main coronary artery (LMCA), left anterior descending artery (LAD), and circumflex artery (CX), associated with extensive calcification. The procedure was performed with the support of the iVAC 2L, and PCI was performed with stenting of the LMCA, LAD, and CX. The entire procedure was performed without the need for vasoactive drugs, and the device was removed without vascular complications.

High-risk PCI has become increasingly common, often requiring ventricular mechanical support. The iVAC 2L offers advantages such as pulsatile flow and ease of implantation, making it an alternative to the intra-aortic balloon pump and Impella. This report highlights the potential use of the iVAC 2L in Brazil, expanding therapeutic options for high-risk patients.

## Introduction

Percutaneous coronary intervention (PCI) is the most commonly performed revascularization method, even in cases of high anatomical complexity, particularly in patients at high surgical risk.^1,2^ However, with the increase in anatomical complexity, associated with population aging and the increased incidence of comorbidities, an increase in the risk profile of PCIs is observed.^3–5^

In this context, mechanical circulatory assistance devices (MCAD) are known to promote improvements in hemodynamic parameters^6.7^ and are recommended for temporary mechanical circulatory support in the high-risk PCI context.^8–11^

The iVAC 2L device (Pulsecath BV, Amsterdam, The Netherlands) is a retrograde insertion MCAD via the femoral artery. The device consists of a bidirectional valve connected to an extracorporeal membrane by a 17Fr cannula. Blood is aspirated from the ventricle through the catheter tip during systole to the membrane, and during diastole, the membrane ejects blood into the ascending aorta through the bidirectional valve (Figure 1). The membrane is moved by mobilizing helium gas through the intra-aortic balloon console (Video 1), which controls the device's operation. The catheter tip is positioned retrogradely below the aortic valve and into the left ventricle (Figure 1). Under ideal conditions (heart rate 70-80 bpm), the additional cardiac output is up to 2.0 L/min, with pulsatile flow. The safety and function of the iVAC 2L have been previously reported.^12,13^

**Figure 1:** 1A) Positioning of the iVAC 2.0 device with the tip of the left ventricle (solid arrow) and the directional valve in the ascending aorta (dotted arrow). A 0.035-inch guide wire helps recognize the position of the aortic sinus (marked with a triangle). 1B) iVAC 2.0 device

**Video 1:** See: http://abccardiol.org/supplementary-material/2025/12210/2025-0236_RC_Video_1.mp4

We will present the first case in Brazil of complex high-risk PCI performed with the support of the iVAC 2L device.

## Clinical Case

We report the case of an 81-year-old man with hypertension and diabetes, referred to our service with dyspnea and class III angina. A functional evaluation with scintigraphy was performed, which demonstrated ischemia in the anterior and anteroseptal walls, in addition to a drop in blood pressure at peak exercise and a left ventricular ejection fraction of 34%. Cardiac catheterization was then indicated, which revealed lesions in the left main coronary artery (80%), left anterior descending artery (70% and 80% lesions in the proximal and middle thirds), and circumflex artery (70% in the proximal third), associated with extensive coronary calcification (Figure 2) and a Syntax Score of 32. The Heart Team discussed the case, and percutaneous treatment was chosen after considering high surgical risk (Euroscore II 7.74%, STS 5.82%), and clinical frailty assessed by a multidisciplinary team.

**Figure 2:** Cardiac catheterization demonstrating lesions of 80% in the left main coronary artery (LMCA), 80% in the LAD, and 70% in the Cx.

The procedure began with femoral puncture, followed by bilateral arteriography (Figure 3) to determine the best strategy. Both common femoral arteries had adequate calibers to accommodate the iVAC 2L device introducer. A significant atherosclerotic lesion was observed in the distal third of the right common femoral artery, and it was decided to position the iVAC 2L device through the left common femoral artery.

**Figure 3:** Left femoral and inguinal arteriography. 3A) Left femoral arteriography demonstrating good caliber of the artery. 3B) Left iliac arteriography demonstrating good caliber of the artery and significant tortuosity in the common iliac artery.

The left femoral artery was pre-closed with two Perclose Proglide devices, followed by insertion of an 18F hydrophilic introducer via a 0.035-inch high-support guidewire ("Amplatz Super Stiff"). Through the right femoral artery, a 0.035-inch guidewire was positioned in the non-coronary sinus to mark the level of the aortic valve. Subsequently, the iVAC 2L device was advanced, positioning its tip in the LV, 2 cm below the aortic valve, maintaining the bidirectional valve in the ascending aorta (Figure 1). The device was connected to the IABP console (intra-aortic balloon pump), configured for ECG triggering, with maximum inflation and 1:1 triggering, providing an estimated additional flow of approximately 2.0 L, given the patient's heart rate of approximately 70-80 bpm (Video 2).

**Video 2:** See: http://abccardiol.org/supplementary-material/2025/12210/2025-0236_RC_Video_2.mp4

We used a 3.5 7Fr EBU catheter for left coronary catheterization. Due to the presence of a severely stenotic lesion, pre-dilation was performed to allow evaluation with intravascular ultrasound (IVUS), which demonstrated a minimum luminal area of 1.88 mm^2^, fibrolipid plaque, and significant calcification, in addition to ulceration in the LMCA and myocardial bridge in the distal segment of the artery (Figure 4). Lesion preparation was performed with dedicated balloons (Cutting Balloon and non-compliant balloon), with adequate results confirmed by IVUS. Subsequently, we implanted drug-eluting stents in the mid-LAD, ostial CX with minimal protrusion into the main, and proximal LAD with extension into the main, using the "Mini-Crush" technique. Post-dilation of the stents was performed, and carina adjustment was performed with the "Kissing-balloon" technique. Control IVUS was performed with adequate expansion and apposition of the struts, without complications (Figure 5). The entire procedure was performed under conscious sedation, without the use of vasoactive drugs. After the procedure, the device was removed, and hemostasis was performed with perclose, as mentioned. Final arteriography showed no vascular complications (Figure 5).^8^

**Figure 4:** Intravascular ultrasound (IVUS) evaluation prior to stent implantation with guidewire positioned in the distal segment of the left anterior descending artery. 4A) Longitudinal section (Long view) from the left anterior descending coronary artery to the left main coronary artery. 4B) Presence of an ulcer in the LMCA, marked with a "#", demonstrating communication between the lesion and the lumen. 4C) Extensive calcification in the left anterior descending artery, with a calcification angle >270° recognized by the posterior acoustic shadow, absent only between 1 and 4 o'clock. 4D) Minimum luminal area in the left anterior descending artery, measuring 1.88 mm^2^. 4E) Presence of a myocardial bridge in the distal portion of the evaluated segment (marked with a "*"), serving as a distal reference for stent positioning. Half-moon sign.

**Figure 5:** Final angiographic and ultrasound results. 5A) Final result with Mini-Crush technique, LCA-DA, and Cx. 5B) Final IVUS evaluation with minimum in-stent luminal area of 6.69 mm2. 5C) Final left femoral arteriography without complications.

## Discussion

High-risk PCIs are becoming increasingly frequent and with better results.^3–5^ In this context, several MCADs were developed.^14^ Historically, IABP has been used in clinical practice for mechanical support in high-risk PCIs. Evidence from randomized clinical trials in this context has failed to demonstrate benefit in reducing major in-hospital cardiovascular events, although long-term analyses indicate a possible, albeit speculative, long-term benefit.^6,15^ Thus, although IABP offers mechanical support and improves coronary and cerebral perfusion, the modest gain in cardiac output and the lack of evidence of cardiovascular benefits led to its non-recommended use in guidelines and consensus.^2,8^

The operation and safety of the iVAC 2L device have been demonstrated in previous studies^12,13,16^ providing a cardiac output of up to 2.0 L/min, approximately 3-4x the output provided by the IABP (Table 1). When compared to continuous flow devices such as Impella and ECMO, its advantages include a possibly more physiological pulsatile flow, ease of implantation, and the use of a balloon console already available in many centers, eliminating the need for a dedicated console. Compared to VA ECMO, iVAC has the advantage, as do Impella and IABP, of decompressing the left ventricle and decreasing systemic vascular resistance. The disadvantages include the 18F profile (compared to the 14F of the Impella CP) and the lower support (up to 2L compared to the 4.3L of the Impella or up to 10L/min of the VA ECMO) (Table 1).

**Table 1:** Comparison between iVAC 2L, IABP, Impella, and VA ECMO devices

	iVAC 2L	BIA	Impella	VA ECMO
Additional debit	2.0L/min	0.3-0.5L/min	2.5-5.5L/min	Up to 7L/min
Access	Femoral 18Fr	Femoral 7-8Fr	13-14Fr	14-19Fr Arterial 17-21Fr ECMO
Mechanism	Extracorporeal membrane	Counterpulsation	Microaxial pump	Centrifugal pump
Synchronization with heart rate	Yes	Yes	No	No
Afterload	Decreases	Decreases	Decreases	Increases

## Data Availability

The underlying content of the research text is contained within the manuscript.
